# 0371. Temporal changes in systemic and renal inflammation and histology in a 72-hour rat model of faecal peritonitis

**DOI:** 10.1186/2197-425X-2-S1-P23

**Published:** 2014-09-26

**Authors:** N Arulkumaran, M Sixma, E Ceravola, R Unwin, FW Tam, M Singer

**Affiliations:** University College London, Bloomsbury Institute of Intensive Care Medicine, London, UK; University College London, Centre for Nephrology, London, UK; Imperial College London, Department of Nephrology, London, UK; Major Hospital Niguarda Ca Granda, Intensive Care Medicine, Milan, Italy

## Introduction

Mechanisms underlying sepsis-induced acute kidney injury remain uncertain with inflammation and altered haemodynamics being implicated.

## Objectives

To determine temporal changes in systemic and renal inflammation, and histology, in a 72 hour fluid-resuscitated model of sepsis.

## Methods

Tunnelled central venous (for fluid administration) and arterial (for BP monitoring/blood sampling) lines were inserted into male Wistar rats under isoflurane anaesthesia. Sepsis was induced 24h later by i.p. injection of faecal slurry. Fluid resuscitation was commenced at 2h post-slurry. Cardiac output was assessed by echocardiography. Animals (n=6-8 per group) were sacrificed at 6, 12, 24, 48, or 72h with kidneys taken for histological section and cytokine (IL-1β, IL-10 by ELISA) analysis, and blood samples for renal biochemistry, and pro- (IL-1β, IL-6), and anti-inflammatory cytokine (IL-10) analysis by ELISA. Renal histology was assessed by light microscopy for features of tubular injury (dilated tubules, tubular casts, cell necrosis), and cell death using TUNEL stain. Comparison was made against sham-operated controls and naïve animals. Statistics were performed using ANOVA and post-hoc Tukey's test. Values are expressed as means±standard deviation.

## Results

Sham animals had values similar to naive animals. In septic animals, serum IL-6 and IL-1β were maximal at 6h; this corresponded with the highest core temperature and tachycardia, and the largest drop in stroke volume. By 24h, serum IL-6 and IL-1β fell, whereas IL-10 levels peaked; this corresponded to recovery of cardiac function and normalization of core temperature. Renal inflammation (peak renal IL-1β and lowest IL-10) was maximal at 24h when serum creatinine also peaked. By 72h, serum creatinine and IL-1β approached baseline values while renal IL-10 reached its zenith. (Table 1) Acute tubular injury was mild and sporadic, with minimal renal tubular cell death.

**Table 1:** Cytokine values

Group	Creatinine (µmol/l)	Serum IL-1β (pg/mL)	Renal IL-1β (pg/µg protein)	Serum IL-10(pg/mL)	Renal IL-10 (pg/µg protein)	Serum IL-6(pg/mL)
Naïve	23±2.6	1.0±0.0	135±36	24±30	207±80	0.8±0.4
6hr Sepsis	26±6	1428±1006	267±46	3390±1759	220±56	36347±27646
12hr Sepsis	21±2.5	1289±296	260±57	2939±1236	89±58	15217±13285
24hr Sepsis	30±5	1434±1601	398±87	4716±2682	180±44	8835±16422
48hr Sepsis	31±4	1008±887	318±140	3699±3745	238±121	144±172
72hr Sepsis	27±5	256±294	157±66	1410±1670	168±37	163±333

## Conclusions

Temporal changes in renal inflammation and dysfunction lag behind changes in systemic inflammation and cardiac dysfunction. Renal dysfunction, as measured by serum creatinine, corresponds to renal inflammation. Cell death does is not a predominant feature, and does not account for the renal dysfunction seen in this sepsis model.

